# Heat Treatment of the Adzuki Bean Borer, *Ostrinia scapulalis* Infected with *Wolbachia* gives Rise to Sexually Mosaic Offspring

**DOI:** 10.1673/031.008.6701

**Published:** 2008-10-31

**Authors:** Hironori Sakamoto, Daisuke Kageyama, Sugihiko Hoshizaki, Yukio Ishikawa

**Affiliations:** ^1^ Graduate School of Agricultural and Life Sciences, The University of Tokyo, Yayoi 1-1-1, Bunkyo-ku, Tokyo 113-8657, Japan; ^2^ National Institute of Agrobiological Sciences, Owashi 1-2, Tsukuba, Ibaraki 305-8634, Japan

**Keywords:** feminization, male killing, sex-specific lethality

## Abstract

A maternally inherited intracellular bacterium, *Wolbachia*, causes reproductive alterations in its arthropod hosts. In the adzuki bean borer, *Ostrinia scapulalis* (Walker) (Lepidoptera: Crambidae), naturally-occurring *Wolbachia* selectively kills male progeny. This *Wolbachia* strain appears to have a feminizing effect, since antibiotic treatment of infected female moths gives rise to male progeny with sexually mosaic phenotypes. It is proposed that male-specific death occurs through the feminizing effect, and sexual mosaics are produced when this effect is incompletely exerted. Here we examined whether the treatment of infected female moths with high temperatures (34°C, 36°C, or 38°C), which is likely to suppress the activity of *Wolbachia*, induces sexually mosaic progeny. It was found that eggs laid within 24 h after treatment of *Wolbachia*-infected mothers at 36°C gave rise to seven sexual mosaics along with 54 normal females. The time lag between treatment and the appearance of mosaic progeny was much shorter with heat treatment than antibiotic treatment, suggesting that heat treatment is more useful for spotting developmental timing when *Wolbachia* exerts its feminizing effect on *O. scapulalis* embryos.

## Introduction

A group of intracellular bacteria belonging to the genus *Wolbachia* (Class Alpha-proteobacteria) have been isolated from a wide variety of arthropods and filarial nematodes (Werren, 1997; Bourtzis and O'Neill, 1998; Stouthamer et al. 1999). Being transmitted exclusively from mothers to offspring, *Wolbachia* increases its fitness by altering the reproductive systems of the host through various means such as cytoplasmic incompatibility, male killing, feminization, induction of parthenogenesis, and others (Werren, 1997; Stouthamer et al. 1999; Charlat et al. 2003).

In the adzuki bean borer, *Ostrinia scapulalis* (Lepidoptera: Crambidae), naturally-occurring *Wolbachia* selectively kill male progeny during embryonic and larval development (Kageyama and Traut, 2004). Interestingly, when *Wolbachia*-infected female moths were treated with tetracycline prior to oviposition, they often produced genetically male moths with sexually mosaic phenotypes (Kageyama et al. 2003a; Kageyama and Traut, 2004). This finding demonstrates that the *Wolbachia* strain in *O. scapulalis* has a feminizing effect on genetic males. Kageyama and Traut (2004) proposed that the feminizing effect underlies the death of *Wolbachia*-infected males, and sexual mosaics are produced when this effect is incompletely exerted.

It is known that in various insect species reproductive manipulation such as cytoplasmic incompatibility, male killing, and parthenogenesis caused by *Wolbachia* can be suppressed by high temperatures, either through direct suppression of the activity of *Wolbachia*, or through the suppression of proliferation of *Wolbachia* in host cells (Trpis et al. 1981; Hoffman et al. 1986; Pintureau et al. 1999; Hurst et al. 2000, 2001). Here we examined whether high temperature treatments of *O. scapulalis* female adults infected with *Wolbachia* can induce the production of sexually mosaic moths.

## Materials and Methods

### Insects

Adult females of *O. scapulalis* were collected at Matsudo, Chiba, Japan, and individually allowed to lay eggs in the laboratory. *Wolbachia* infection in collected females was checked using a diagnostic polymerase chain reaction (PCR) assay using primers specific for *Wolbachia* 16S ribosomal DNA (O'Neill et al. 1992) as described previously (Kageyama et al. 2003b). The offspring of single, *Wolbachia*-infected and uninfected females were reared to adults on a commercial artificial diet (Silkmate™ 2M, Nosan Corp, www.nosan.com) at 25°C, under a 16L: 8D. These insects were used to establish a *Wolbachia*-infected matriline (MDOO 14; Kageyama and Traut, 2004) and two uninfected matrilines (MD2161 and MD2164).

### Heat treatment

Thirty *Wolbachia*-infected or -uninfected female moths were allowed to mate with 30 uninfected males in a screen cage (20 cm × 20 cm × 20 cm) for two days. After males were removed, the cages were transferred into incubators maintained at 25°C (control), 34°C, 36°C or 38°C for two days, and survival rates were then examined. Surviving female moths were individually transferred into plastic cups (10 cm in diameter, 4.5 cm in depth) to allow oviposition at 25°C under 16L: 8D. Eggs were collected daily, and larvae were reared by broods and oviposition dates. The pupae produced were sexed by examining the morphology of the abdominal terminal segment and midleg (males have larger mid-tibiae than females) under a stereomicroscope, and then maintained separately in plastic cups with a piece of moistened filter paper.

## Results

The matriline MD0014 exhibited the all-female trait, which was shown to be due to the *Wolbachia*-induced male killing (Kageyama and Traut, 2004). In matrilines MD2161 and MD2164, sex ratios did not significantly deviate from 1:1.

The survival rates of females after heat treatment decreased as temperature rose (Table 1). No significant difference in survival rates were detected between infected and uninfected females at any temperature examined (P > 0.05, 2 × 2*χ*^2^ test).

**Table 1.**
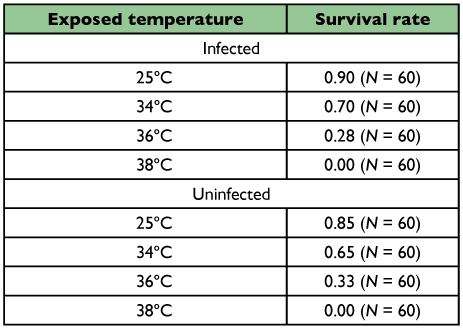
Survival rates of female *Ostrinia scapulalis* after heat treatment

No effect of high temperature treatments on the sex ratio or sex differentiation was observed in the progeny of uninfected mothers; they consistently developed to normal males and females at nearly 1:1 ratio (Table 2). In contrast, progeny of the infected mothers treated at 25°C and 34°C developed to only female adults (Table 2). Among the progeny of infected mothers treated at 36°C, seven of 97 individuals examined showed sexually mosaic phenotypes at the pupal stage (Table 2; Figure 1A). The seven mosaic pupae had male-like mid-tibia and femalelike morphology in the last abdominal segment (Figure 1A). All seven mosaic pupae were derived from eggs laid within 24 h after the completion of heat treatment. Two of the mosaic pupae developed to adults, and the other five died. The wings of the two emerged moths, although curled, exhibited wing mark/color patterns characteristic of the sexual mosaics (Figure 1D). The curled wings were observed only in sexual mosaics. Mosaic patterns were also found in the coloration of the dorsal abdomen of the two individuals (Figure 1D). Except for these sexual mosaics, all progeny produced by *Wolbachia*-infected females consisted of normal females. Almost all the offspring of infected mothers maintained at 25°C (control) developed to adults, > 0.97 in most cases.

**Table 2.**
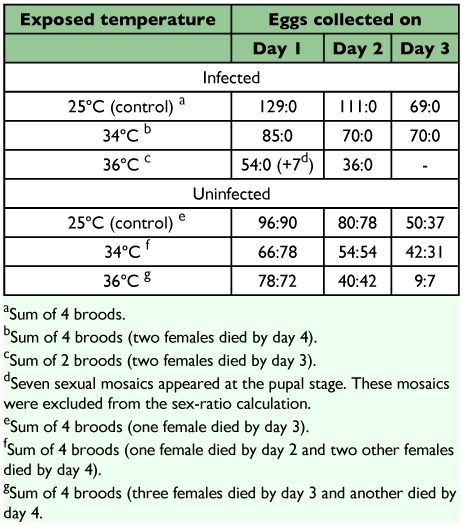
Sex ratio (female: male) of the progeny of *Wolbacha*-infected and -uninfected *Ostrinia scapulalis* collected daily as eggs after heat treatment.

## Discussion

In the present study, sexually mosaic progeny were produced from *Wolbachia*-infected *O. scapulalis* females treated with a high temperature (36°C) prior to oviposition. Thus, two different treatments of *Wolbachia*-infected *O. scapulalis* female moths, i.e., high temperature and oral administration of antibiotics (Kageyama et al. 2003a), have been shown to induce sexually mosaic moths. The present finding confirmed that *Wolbachia* in *O. scapulalis* has the potential to interfere with the host's sex determination pathway.

Heat and antibiotic treatments of infected *O. scapulalis* females showed a clear difference in the induction pattern of sexually mosaic moths, as follows. In antibiotic treatment, sexual mosaics developed from eggs laid ≥ 4 days after treatment in *O. scapulalis* (Kageyama et al. 2003a; Kageyama and Traut, 2004), and ≥ 3 days after treatment in the sister species *O. furnacalis* (Sakamoto et al. 2007). Meanwhile, the effect of heat treatment appeared promptly and transiently; sexual mosaics developed from eggs laid < 24 h after treatment, but no sexual mosaics developed from eggs laid > 24 h after treatment. It is possible that orally administered antibiotics take longer to show an effect because they must be absorbed through the gut and then transported to the reproductive tissue. The transience of the effect of high temperature suggests that the occurrence of sexual mosaics is a consequence of direct suppression of the activity of *Wolbachia*, rather than the consequence of an indirect effect such as a decrease in *Wolbachia* density.

Regarding the generation of sexually mosaic moths, we hypothesize that *Wolbachia* interferes with key step(s) of sex determination during the egg/embryonic development of individuals with the male genotype (ZZ). Considering the promptness and transience of the effect, heat treatment would be more useful than antibiotic treatment for spotting crucial time events when *Wolbachia* interferes with the sex determination step(s) during development. Heat treatments at various times during egg/embryonic development would clarify the crucial timing of *Wolbachia* action against normal sex determination in genetic males of *O. scapulalis.* Such knowledge may give insights into the mechanism of *Wolbachia*-induced feminization in arthropods.

Finally, only two moths successfully emerged from seven sexually mosaic pupae. The death of five mosaic pupae might be attributable to the delayed expression of male killing; heat treatment probably decreased the *Wolbachia* density to a level at which it could not kill male hosts during the larval stage, but *Wolbachia* density may have subsequently recovered to a level sufficient to kill male hosts at the pupal stage. It is known in the butterfly *Hypolimnas bolina* that tetracycline treatment of females leads to the attenuated and delayed expression of male killing (Mitsuhashi et al. 2004; Charlat et al. 2007). In *H. bolina*, *Wolbachia* has the ability to kill males during the larval stage through bacterial activity (Charlat et al. 2007).
